# Alveolar ridge preservation in post-extraction sockets using concentrated growth factors: a split-mouth, randomized, controlled clinical trial

**DOI:** 10.3389/fendo.2023.1163696

**Published:** 2023-05-17

**Authors:** Sadam Ahmed Elayah, Hamza Younis, Hao Cui, Xiang Liang, Karim Ahmed Sakran, Baleegh Alkadasi, Essam Ahmed Al-Moraissi, Mohammed Albadani, Wafa Al-Okad, Junbo Tu, Sijia Na

**Affiliations:** ^1^ Key Laboratory of Shaanxi Province for Craniofacial Precision Medicine Research, College of Stomatology, Xi’an Jiaotong University, Xi’an, China; ^2^ Department of Oral and Maxillofacial Surgery, College of Stomatology, Xi’an Jiaotong University, Xi’an, China; ^3^ State Key Laboratory of Oral Diseases and National Clinical Research Centre for Oral Diseases and Department of Oral and Maxillofacial Surgery, West China Hospital of Stomatology, Sichuan University, Chengdu, China; ^4^ Department of Oral and Maxillofacial Surgery, College of Dentistry, Ibb University, Ibb, Yemen; ^5^ Department of Oral and Maxillofacial Surgery, College of Dentistry, Thamar University, Thamar, Yemen; ^6^ Department of Oral and Maxillofacial Surgery, College of Dentistry, Sana’a University, Sana’a, Yemen

**Keywords:** platelet, impacted teeth, extraction, alveolar ridge, regeneration, CGF

## Abstract

**Aim:**

The aim of this clinical trial was to assess the impact of autologous concentrated growth factor (CGF) as a socket-filling material and its ridge preservation properties following the lower third molar extraction.

**Materials and methods:**

A total of 60 sides of 30 participants who had completely symmetrical bilateral impacted lower third molars were enrolled. The primary outcome variables of the study were bone height and width, bone density, and socket surface area in the coronal section. Cone beam computed tomography images were obtained immediately after surgery and three months after surgery as a temporal measure. Follow-up data were compared to the baseline using paired and unpaired *t*-tests.

**Results:**

CGF sites had higher values in height and width when compared to control sites (Buccal wall 32.9 ± 3.5 vs 29.4 ± 4.3 mm, Lingual wall 25.4 ± 3.5 vs 23.1 ± 4 mm, and Alveolar bone width 21.07 ± 1.55vs19.53 ± 1.90 mm, respectively). Bone density showed significantly higher values in CGF sites than in control sites (Coronal half 200 ± 127.3 vs -84.1 ± 121.3 and Apical half 406.5 ± 103 vs 64.2 ± 158.6, respectively). There was a significant difference between both sites in the reduction of the periodontal pockets.

**Conclusion:**

CGF application following surgical extraction provides an easy, low-cost, and efficient option for alveolar ridge preservation. Thus, the use of CGF by dentists during dental extractions may be encouraged, particularly when alveolar ridge preservation is required.

**Clinical trial registration:**

TCTR identification, TCTR20221028003.

## 1 Introduction

One of the most critical competencies in advanced dentistry is alveolar ridge preservation (ARP) after exodontia. The loss of alveolar bone may be attributed to a variety of factors (1), including aggressive extraction procedures, periodontal disorders, tumors, infections, or cysts (2). Exodontia is a traumatic procedure that often results in the destruction of soft tissue and alveolar bone. During wound healing, a complex cascade of anatomical and physiological processes takes place in the architecture of the soft tissue and alveolar bone destruction (3), which occurs during the first three months (1).

The mechanism of extraction socket healing is characterized by internal changes that result in the formation of the bone inside the socket and external changes that result in the reduction in the height and width of the alveolar ridge (1). In 1985, the ARP after exodontia was first described by Greenstein (1). The demand for ARP has highly increased recently. Thus, several approaches have been investigated in an attempt to suppress bone resorption and preserve the shape of the dental socket (4). The majority of bone grafting studies encounter various challenges (5). One of the most modern techniques is to hasten the healing of bone grafts by stimulating growth factors, which are bioactive proteins that govern the wound healing and bone regeneration processes (6).

Platelets are a major source of autogenous growth factors (7). According to their properties and preparation techniques, platelet substitutes can be categorized into three generations (8). Platelet-rich plasma (PRP), the first generation, was established in the 1970s. The second generation is platelet-rich fibrin (PRF); its first introduction was in 2001 (9). Concentrated growth factors (CGF) are the third and most recent generation of platelet substitutes established by Sacco in 2006 (10); it is a novel concentrated platelet substance that is used to repair bony defects and enhance the success of bone grafting techniques (11). It is obtained from the fresh venous blood of the individual without anticoagulants through centrifugation, immediately using special centrifuge equipment (12). CGF has a significant effect on postoperative complications such as delayed wound healing, swelling, and pain after surgical extraction (12).

At present, there is only one publication on the implications of CGF combined with bone graft materials on alveolar ridge preservation (13). Thus, the application of CGF is limited to the available scientific evidence. To better comprehend the features and therapeutic application of CGF, more basic and randomized clinical trial studies should be done (12, 14, 15). In addition, most CGF trials were concerned with short-term clinical outcomes (12, 16–18), and no long-term, split-mouth radiographic studies of the usage of CGF alone have been published, to the best of our knowledge. Therefore, the purpose of this randomized clinical trial was to evaluate the effectiveness of CGF in alveolar ridge preservation. We used the lower third molar region as a study model. The research question is: When compared to natural healing, does using CGF as a socket-filling substance result in a satisfied alveolar ridge preservation after dental extraction?

## 2 Material and methods

**Figure 1:**
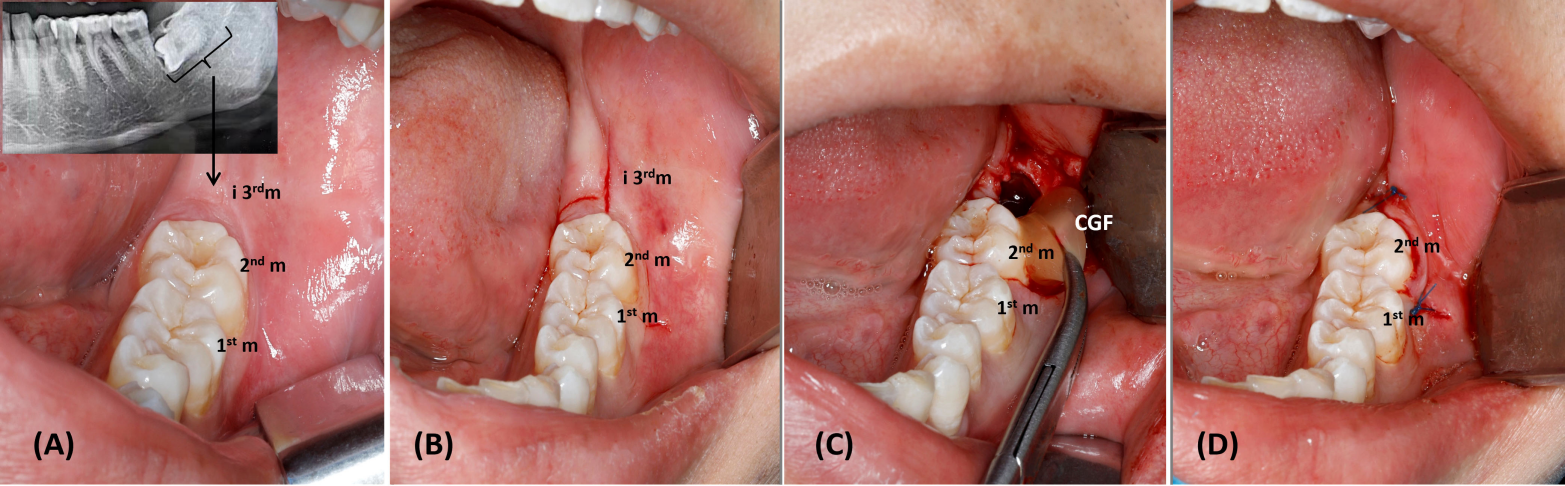
Lower third molar extraction. **(A)** Impacted Lower third molar, 1st m: first molar, 2nd m: second molar, and i3rd m: impacted third molar. **(B)** Modified Ward’s Incision, 1st m: first molar, 2nd m: second molar, and i3rd m: impacted third molar. **(C)** Placement of CGF in the extracted sockets, 1st m: first molar, 2nd m: second molar, and CGF; concentrated growth factors. **(D)** Wound suturing, 1st m: first molar, 2nd m: second molar.

This prospective, split-mouth, randomized, single-blind, clinical study was conducted in accordance with the Helsinki Declaration at the outpatient clinic department of oral surgery-hospital of Stomatology, Xian Jiaotong University from 25 June 2022 to 20 October 2022. This trial protocol was reviewed and approved by the Hospital of Stomatology’s institutional ethics committee at Xian Jiaotong University, in Xian, China, (xjkqII[2022] No: 033). In addition, it was registered with the TCTR identification number TCTR20221028003 at the Thai Clinical Trials Register-Medical Research Foundation of Thailand and written informed consents were obtained from all participants. For this study, a total of 60 sides of 30 patients (16 men/14 women) within the age group of 19 to 35 years (average age of 25 years) were included in the study. The following demographic and clinical characteristics were collected: age, gender, and impaction type **(** **Table S1**). Before surgery, all patients had a physical and radiographic evaluation. The patients were selected based on the following criteria: (a) age ≥18; (b) patients with completely impacted lower third molars (**Figure 1A**) that are symmetrically, bilaterally, horizontally, or vertically positioned with a difficulty index ranging from 7 to 10 based on Pederson’s description (19) in need of surgical extraction; (c) no pericoronitis or periapical lesions; (d) patients who are cooperative and able to attend the follow-up visits; (e) neither a history of systemic diseases nor the use of systemic drugs. All of the patients had been notified of the treatment plan and the study’s objectives, and they had undertaken surgical extraction of both of their impacted lower third molars in a single visit (12, 20).

### 2.1 Sample size calculation

The sample size was determined using the G*power 3.0.10 software. The required minimum sample size was 24 subjects for each group. This demonstrated that a target significance value of 0.05 would need a sample size of 30 subjects (30 test sides and 30 control sides) to have 85% power to detect a statistical difference between the CGF and control sites. Additionally, it was conducted consistent with previous comparable studies (1, 21, 22).

### 2.2 CGF preparation

**Figure 2:**
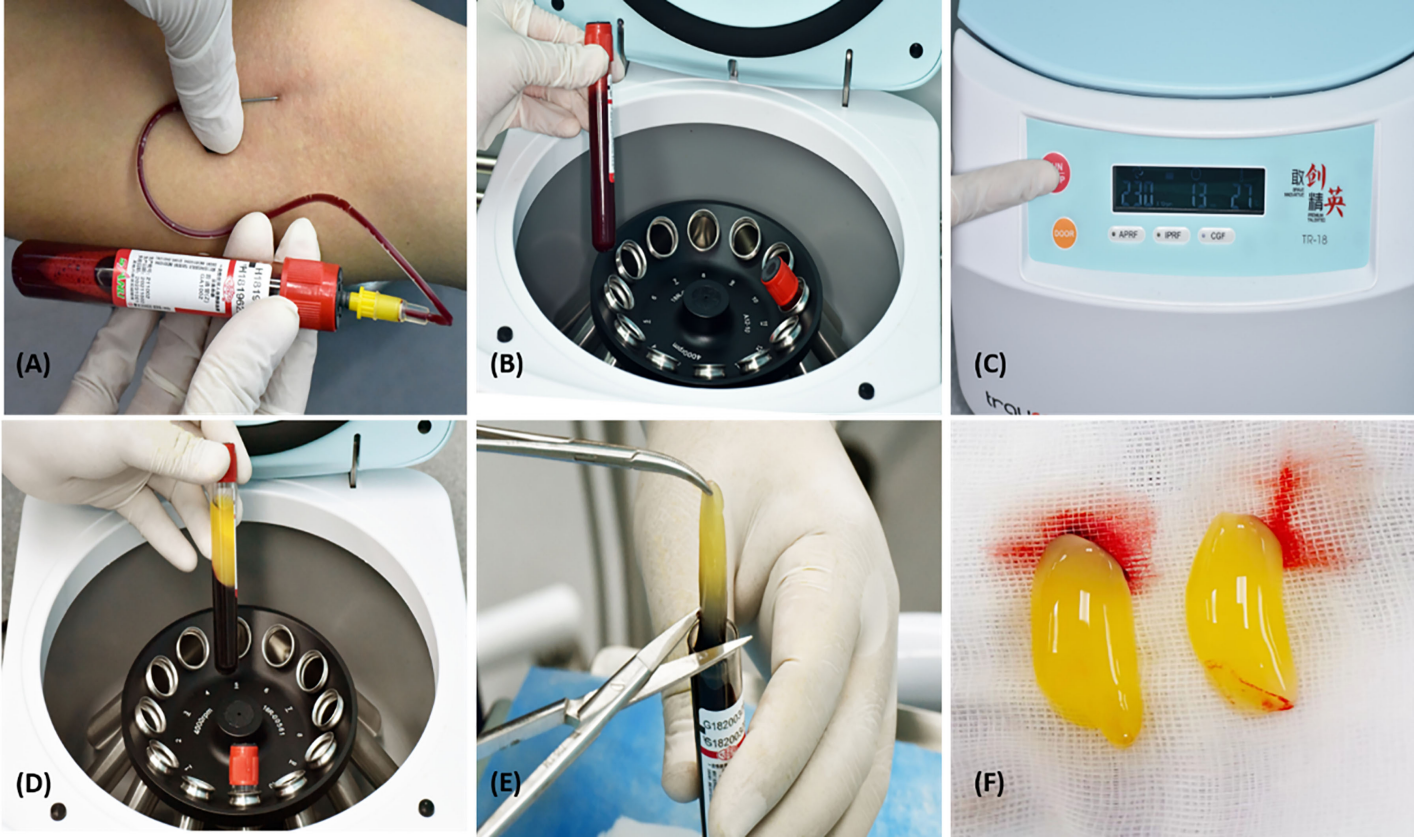
Preparation of CGF. **(A)** Blood withdrawal, **(B)** Two Sterilised 10 ml tubes, **(C)** CGF centrifuge equipment, trausim, **(D)** After centrifugation, **(E)** Blood clots are removed from the CGF fibrin using scissors, and **(F)** CGF fibrin gel.

Fresh venous blood from patients was used to collect autologous CGF samples. They were divided into two clean 10 ml tubes without adding any anticoagulants, and they were centrifuged immediately (10) using a CGF centrifuge machinery (Trausim, DL4015, Dental Regenerative Centrifuge, China) (12) according to the following guidelines: running time: 13 minutes; temperature in the chamber: 21; speed: 230*10rpm. Each CGF clot was taken out of the tube after centrifugation and split from the red element phase using scissors (**Figure 2**) (21).

### 2.3 Randomization

An opaque, sealed envelope (12, 21, 23) picked by the patient was used for randomization to choose the side on which CGF was to be placed. Cards labeled “R” or “L” inside the envelopes indicated the surgical site to receive CGF. A nurse who was not engaged in the trial sealed and then reopened them after the patients made their choice. Outcome assessors (Elayah, Younis, and Cui) were not aware of the CGF site. Consequently, this trial was an assessor-blind trial (23). The same protocol was followed for all surgeries by the same experienced surgeon.

### 2.4 Study variables

The application of CGF is regarded as the study’s main variable.

The primary outcome variables of the study were bone height and width, bone density, and socket surface area.

The secondary outcome variable was the periodontal pocket of the second molars.

### 2.5 Surgical procedure

The same surgical protocol as in our previous study (12) was followed for all surgical teeth extractions with the following steps:

- Each patient had a radiologic examination, including a panoramic radiograph before surgery (**Figure 1A**).
- Blood was collected from patients.
- Patients gargled with 0.12% chlorhexidine gluconate as an antiseptic mouthwash for one minute.
- The root surfaces of lower second molars were scaled and root planed to achieve a smooth surface. Then, CGF fibrin gel was injected into the second molar pockets of CGF sites.
- Modified Ward’s Incision (24) was used under local anaesthesia (**Figure 1B**).
- We performed bone removal, tooth separation and extraction, and socket irrigation.
- We placed CGF in the extracted sockets of CGF sites which were randomly selected (**Figure 1C**).
- We allowed natural healing to occur after extraction in control sites.
- We sutured the wound for both sockets (**Figure 1D**).
- We prescribed antibiotics, anti-inflammatory analgesics, and antibacterial mouthwash.
- We gave patients instructions for postoperative care.
- One week following the surgery, the sutures were removed, and the wound was gently irrigated with saline.
- We carried out a postoperative follow-up on the 7^th^ day and 3^rd^ month.

### 2.6 Clinical features assessment

The assessment of the periodontal pockets of the second molars was done before surgical extraction and after 3 months using the UNC-15 periodontal probe (25). In this context, the periodontal pockets were diagnosed as simple, compound, or complex depending on the number of surfaces involved (26, 27).

### 2.7 Radiographic features assessment

**Figure 3:**
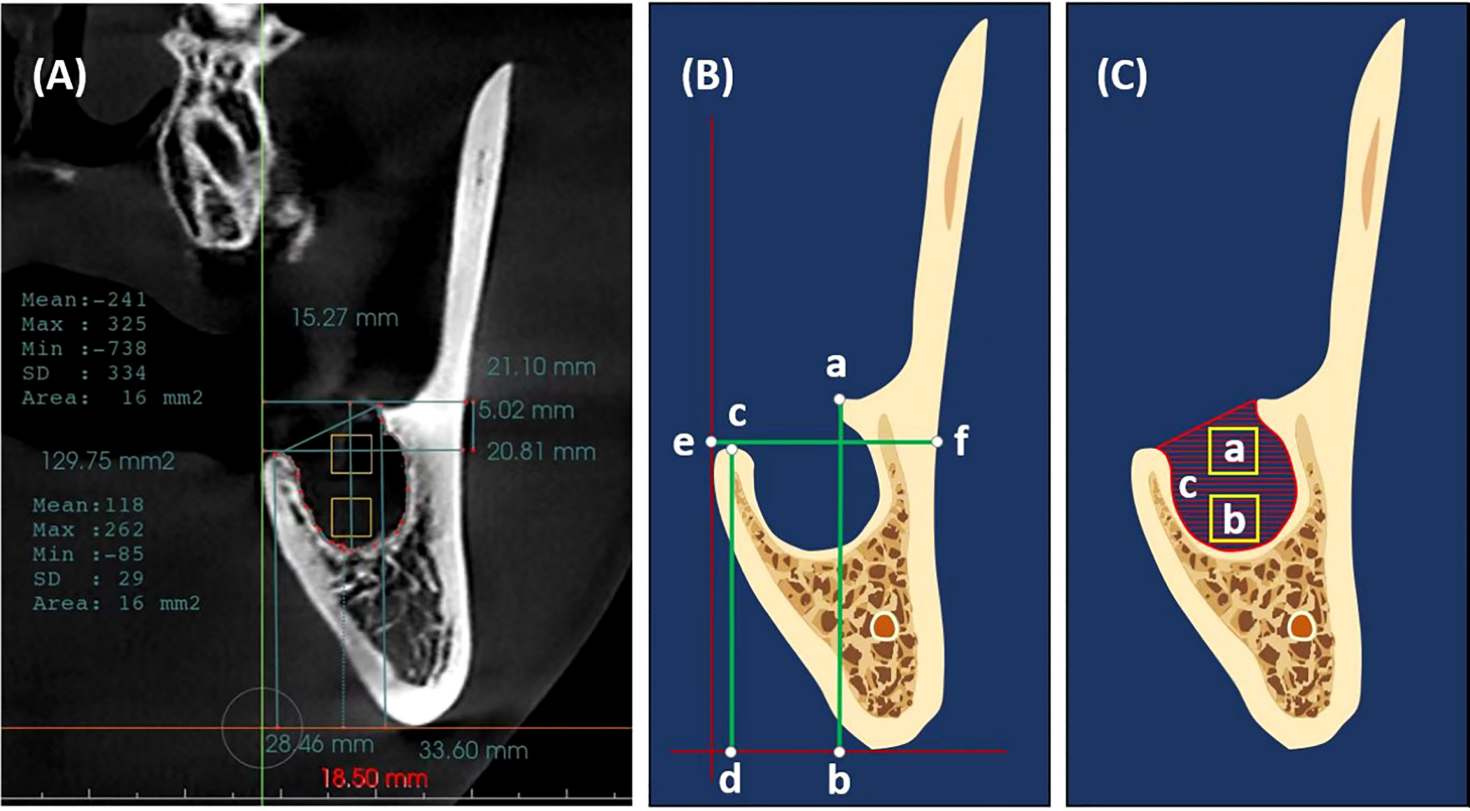
Measurements methodology **(A)** A cone beam computed tomography (CBCT) scan immediately after extraction with Invivo Dental showing the measurement methodology. **(B)** Measurements of buccal alveolar bone height (a, b), lingual alveolar bone height (c, d), and alveolar bone width (e, f). **(C)** Measurements of bone density (a, b squares in the coronal and apical halves of the extracted socket) and socket surface area (c, Hatch area); the outline of the remnant socket.

A cone beam computed tomography (CBCT) was taken immediately after extraction (C1), followed by a CBCT three months later (C2) as a temporal measure (1, 21, 28, 29). For (C1 and C2), Invivo Dental 5.0 (Anatomage Inc., San Jose, CA, USA) was used for radiographic assessment (**Figure 3A**) (30). A coronal section at the approximate midpoint of the extraction socket was used for measuring buccal and lingual bone heights, alveolar ridge width, and socket surface area, as well as bone density in the coronal and apical halves of the socket. Stable anatomical landmarks (anterior nasal spine, mental foramen, infraorbital foramen, lower border of the mandible, and genial tubercle) along with superimposition were used to ensure the accuracy of measurements at the same section in both CBCTs (1).

Buccal and lingual alveolar bone heights were measured as the vertical distances between the horizontal tangent line of the lower border of the mandible and the crest of the buccal and lingual socket walls, respectively (31) **(** **Figure 3B** and **Supplementary Figure 1**). Alveolar bone width was measured as the bucco-lingual distance between the outer buccal and lingual borders of the alveolar ridge at the level of the extraction socket coronally **(** **Figure 3B**).

Bone density was measured as the mean Hounsfield units (HU) within a 16mm^2^ area (32) in the coronal and apical halves of the extraction socket; the socket was divided into two halves apicocoronally, and the measurement square was placed in the center of each half **(** **Figure 3C**). The socket surface area was measured along the inner bony border of the socket in the same coronal section **(** **Figure 3C**).

### 2.8 Data analysis

The Statistical Package for Social Sciences (SPSS) version 25 (Chicago, USA) was used to compute descriptive and analytical statistics. Paired *t*-test was used to compare the buccal wall, lingual wall heights, and width of the alveolar bone at different time intervals as intra-site, and for inter-site comparisons, an unpaired t-test was used. The bone density and socket surface area were compared using the Mann-Whitney test. The periodontal pocket index was calculated at both sites using the chi-square test. Furthermore, the intraclass correlation coefficient test (ICC) was used to assess the intra-observer reliability of socket dimensions (**Table S2**). The significance was regarded as *P* < 0.05, while highly significant as *P* < 0.001.

## 3 Results

In this clinical trial, 30 patients (16 men and 14 women) with an average age of 25 years had surgical extraction of both of their impacted lower third molars in a single visit at the outpatient clinic department of oral surgery-Hospital of Stomatology, Xian Jiaotong University. We used the lower third molar region as a study model. In terms of resorption, the buccal wall, lingual wall heights, and alveolar bone width showed a statistically significant reduction in the height and width of control site C2 when compared to baseline C1 of the same site (C1 31.4 ± 4.5 vs C2 29.4 ± 4.3 mm, C1 26.4 ± 4.2 vs C2 23.1 ± 4 mm, C1 20.51 ± 1.82 vs C2 19.53 ± 1.9 mm, respectively), while CGF sites C2 showed a significant increase in the wall height and width (C1 30.9 ± 3 vs C2 32.9 ± 3.5 mm, C1 23.7 ± 3.7 vs C2 25.4 ± 3.5 mm, C1 20.87 ± 1.61 vs C2 21.07 ± 1.55 mm, respectively). The CGF site had higher values in height and width when compared to the control site (Buccal wall *P*= .002, Lingual wall *P*= .003, and Alveolar bone width *P*<.001).

The intra‐ and inter‐sites comparisons are shown in (**Tables 1a–c** **).** In terms of osteogenesis, measurements of bone density in the coronal and apical halves showed that a significantly higher proportion of individuals had higher values in the CGF sites compared to the control sites (Coronal half 200 ± 127.3 vs -84.1 ± 121.3, Apical half 406.5 ± 103 vs 64.2 ± 158.6) **(** **Tables 2a, b** **).** Meanwhile, the CGF sites showed a statistically significant reduction in the socket surface area (*P*=.001) **(** **Table 2c** **).** There was a significant difference between both sites regarding the reduction of the periodontal pocket, that is, the CGF side had significantly less periodontal pocket than the control side (P<.001) (**Table 3** **).**

**Table 1:** a, b & c: Comparison of Buccal and lingual walls height and alveolar bone width in terms of mean±standard deviation at different time intervals in both sites.

Variables	CGF site	Control site	P_2_
(a) Buccal wall height (mm)			
Immediately after surgery Mean±SD	30.9±3	31.4±4.5	.62*
3^rd^ month after surgery Mean±SD	32.9±3.5	29.4±4.3	.002**
** *P_1_***	<.001	<.001	
(b) Lingual wall height (mm)			
Immediately after surgery Mean±SD	23.7±3.7	26.4±4.2	.014*
3rd month after surgery Mean±SD	25.4±3.5	23.1±4	.003**
*** P_1_***	<.001	<.001	
(c) Alveolar bone width (mm)			
Immediately after surgery Mean±SD	20.87±1.61	20.51±1.82	.42*
3rd month after surgery Mean±SD	21.07±1.55	19.53±1.90	<.001**
*** P_1_***	<.001	<.001	

P_1=_ P-value of one site (intra-site) using paired t-test, P_2_= P-value of both sites (inter-site, * means P-value of immediately after surgery, ** means P-value of 3^rd^ month after surgery) using unpaired t-test, mm; millimetre.

**Table 2:** a, b & c: Comparison of bone density in the cervical and apical thirds of the socket and socket volume using Mann –Whitney test.

(a) Bone density	Immediately after surgery		3^rd^ month after surgery	
	Mean (SD)	Median (Max-Min)	Mean (SD)	Median (Max-Min)
**Coronal half** CGF site	-200.6 (203.2)	-180 (-614-296)	200 (127.3)	197 (-53 - 475)
Control site	-188.6 (169)	-233 (-542 - 240)	-84.1 (121.3)	-104 (-297-250)
***P*-value**	.901		< .001	
**(b) Apical half** CGF site	-109.4 (186.6)	-124 (-524-246)	406.5 (103)	420 (111- 623)
Control site	-134.9 (152)	-169 (-323-281)	64.2 (158.6)	65 (-155-434)
***P-*value**	.465		< .001	
**(c)** S**ocket surface area** CGF site	121 (27.1)	120.4 (81.2-192.6)	31.1 (19)	24.7 (4.2- 76.4)
Control site	101.5 (37.2)	94.5 (42.2 - 163.6)	59.9 (35.8)	48.2 (3 -119.6)
***P-*value**	.032		.001	

**Table 3:** Comparison of periodontal pocket of second molar at different time intervals using Chi-Square test.

	Periodontal Pocket index				
	Normal (0)	Simple (1)	Compound (2)	Complex (3)	*P_2_*
CGF site					
Immediately after surgery	2	10	18	0	
3^rd^ month after surgery	25	5	0	0	.150*
***P_1_***		<.001			
Control site					
Immediately after surgery	2	10	18	0	
3^rd^ month after surgery	4	17	9	0	<.001**
***P_1_***		<.001			

P_1=_ P-value of one site (intra-side) using paired t-test, P_2_= P-value of both sites sides (inter-site, * means P-value of immediately after surgery, ** means P-value of 3^rd^ month after surgery) using Chi-square test.

## 4 Discussion

The 1980s saw the introduction of bone grafts in fresh sockets for ARP (33). Numerous ARP techniques have been developed with time, employing various bone graft substances including autografts, allografts, xenografts, alloplasts, and hybrids of these substances (34). Contrasted to the use of autologous platelet concentrates (APCs) techniques, the use of autograft bone has disadvantages, including the necessity for a second surgical site, donor site mortality rates, postoperative pain, an additional operating time and cost, an expanded danger of donor site fracture, and a limited amount of graft depending on the donor site selected (35). Allografts, xenografts, and alloplasts encounter a multitude of issues, including a high risk of disease transmission, autoimmune rejection, infection, residual graft substances, and a long healing process, as well as their high cost (5). Consequently, several growth factor studies have shown that autologous growth factors, which have been clinically proven to stimulate tissue regeneration, are the best tissue regenerative stimulus (12).

Thus, novel bio-active methods have been devised to counteract the limitations of previous bone graft substances. Autogenous growth factors are mostly derived from platelets. Growth factors such as PRP, PRF, and CGF are bioactive proteins that regulate the mechanism of bone and soft tissue regeneration (12).

**Figure 4:**
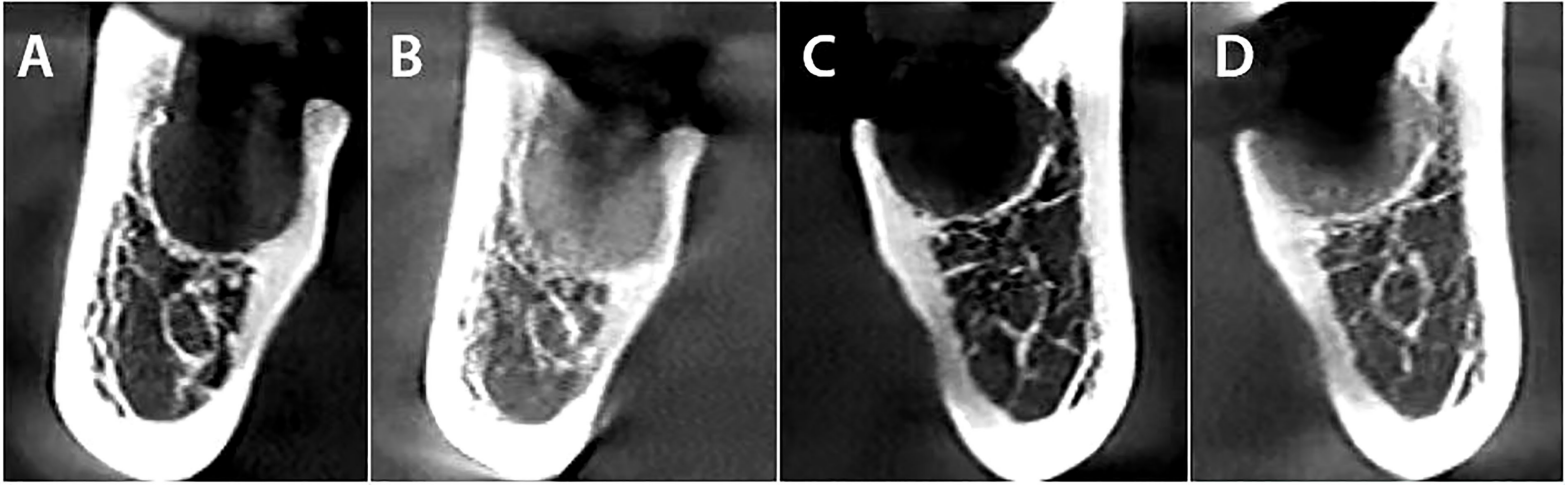
The comparison between both sides immediately after extraction, followed by a CBCT three months later. **(A)** CGF side immediately after extraction. **(B)** CGF side three months after the dental extraction. **(C)** Control side immediately after extraction. **(D)** Control side three months after the dental extraction.

The current prospective, split-mouth, randomized, controlled clinical trial confirmed that sockets grafted with CGF had better preservation of the alveolar ridge and a reduction of periodontal pocket depth, compared to natural healing (**Figure 4** **)**.

In terms of bone resorption, the present findings showed significant differences (P<.001) for resorption between C1 and C2 in the control site; buccal wall, lingual wall heights, and alveolar bone width showed a statistically significant reduction in the control site C2 when compared to baseline C1 of the same site, while CGF sites showed a significant increase of the wall heights and width. This emphasizes the outcomes of previous studies, one of which reported that the application of CGF may reduce both vertical and horizontal bone resorption after posterior tooth extraction (36). Chen et al. (5) demonstrated that CGF is a superb cell growth factor biomaterial that has a very positive influence on osteogenesis and angiogenesis. Sohn et al. (37) found that using fibrin-rich blocks with CGFs instead of bone grafting and simultaneous implanting showed successful new bone regeneration in the maxillary sinus. When we compared our CGF findings to previous studies of PRF, Al-Hamed et al. (38) in a Systematic Review and Meta-Analysis study found that following the removal of the mandibular third molars, PRF appears to have no favorable effect on bone healing. As reported in Srinivas’s study (1), there was no significant difference between the alveolar bone height of the PRF group and the control group. Similarly, Zhang et al. (39) reported that preserving the alveolar ridge with PRF alone has no significant effect on minimizing bone resorption. Girish Kumar et al (40) compared three groups: group A sockets were chosen as control, group B sockets were grafted with PRF, and group C sockets were grafted with Plaster of Paris as a bone substitute and then covered with PRF. He reported that there was no statistically significant difference in ridge resorption among the three groups. By contrast, Doiphode et al. (41) concluded that alveolar ridge resorption may be reduced, and bone formation may be promoted by PRF.

In terms of bone density, Ma et al. (36) reported that the CGF group had better results in bone mineral density and the microarchitecture of the trabecular bone compared with the control group. Manoj S et al. (42) observed that a significant increase in the bone volume and density of the newly formed bone was evident in implants that were immediately placed with CGF grafting. These findings are in agreement with our findings which concluded that a significantly higher proportion of individuals had higher bone density values in the CGF site when compared to the control site (P < .001); meanwhile, CGF sites showed a statistically significant reduction in the socket surface area (P=.001).

Jung et al. (43) radiographically compared four approaches for ridge preservation after tooth extraction (β-tricalcium-phosphate-particles with polylactid coating, demineralized bovine bone mineral with 10% collagen and a collagen matrix (DBBM-C/CM), DBBM-C covered with an autogenous soft-tissue graft, and spontaneous healing as a control group) and concluded that the alveolar ridge could not be completely preserved using any of the approaches.

Based on a slew of studies, traditional therapy for the extraction of impacted third molars often leads to the development of an osseous defect at the distal aspect of the second molar, which may need a surgical repair later (44, 45). Consequently, CGF was shown to be a reliable aid in the reduction of periodontal intrabony pockets, and CGF is often employed in implant and periodontal surgery as well as gingival repair or regeneration alone or in combination with several biological substances (25). Li et al. (46) concluded that CGF enhances human periodontal ligament cells (hpdlcs) osteogenesis in a tumor necrosis factor alpha-induced inflammatory microenvironment in addition to having an osteogenic impact (hpdlcs) in normal culture. Bozkurt et al. (47) suggested that the use of CGF in combination with a coronally advanced flap may increase the success of gingival recession treatment. These studies agreed with the findings of the present study; there was a significant difference between both sites regarding the reduction of the periodontal pocket; the CGF side had significantly less periodontal pocket than the control side (P<.001).

In summary, compared to PRP and PRF, CGF contains more growth factors (37, 48). Additionally, CGF has a complex internal structure which can affect the release of growth factors, metabolites, and cells. These cells might control the synthesis and release of the CGF growth factors, exhibit stem-like characteristic features, and have the capability of differentiation into osteoblasts, which generate a mineralized matrix (8). The limitations of the present study include the small sample size and the lack of histological evidence.

## 5 Conclusion

Extracted sockets grafted with CGF showed a good and successful outcome with regard to alveolar ridge preservation. CGF application following surgical extraction provides an easy, low-cost, and efficient option for alveolar preservation, considering its biocompatibility, resilience, and availability. Thus, the use of CGF by dentists during dental extractions may be encouraged, particularly when alveolar ridge preservation is required.

## Data availability statement

The original contributions presented in the study are included in the article/**Supplementary Material**. Further inquiries can be directed to the corresponding authors.

## Ethics statement

The study protocol was reviewed and approved by the institutional ethics committee at the Hospital of Stomatology, Xian Jiaotong University, Xian, China. The patients/participants provided their written informed consent to participate in this study.

## Author contributions

SE, HY, and HC contributed to data collection, interpretation of data, designing the study, and writing the original manuscript. All other authors have critically revised the manuscript and have approved the final manuscript prior to its submission.
