# Supplementary material for: Alveolar ridge preservation in post-extraction sockets using concentrated growth factors: a split-mouth, randomized, controlled clinical trial
Source: Front Endocrinol (Lausanne). 2023 May 17;14:1163696. doi: 10.3389/fendo.2023.1163696 (PMC10231034; doi:10.3389/fendo.2023.1163696)

**S.Fig.1;** The reason of why the bone height in our study was measured as the vertical drop line from the highest point of the crest towards a horizontal line that is on the level of the lowest point at the inferior border:

The reason is that after bone remodeling, the highest point at the crest might go more buccally or lingually compared to the original point, if the height was measured as the straight line between this point and the lower border, the line before and the line after healing will have different angles, which may incorrectly represent the change in bone height. In the following illustration **(SF.1)**, the green highlighted area represents the alveolar bone after healing, we can see that line A and line B have equal length but different angles, which may conclude that bone height did not change. But if a vertical drop line was used, we can see that line C and line D are not equal, and that is a better way to represent change in height without angles influencing the measurement**.** Our method better describes the change in vertical bone height rather than the distance between the crest and the lower border which is influenced by the angle between the two points.


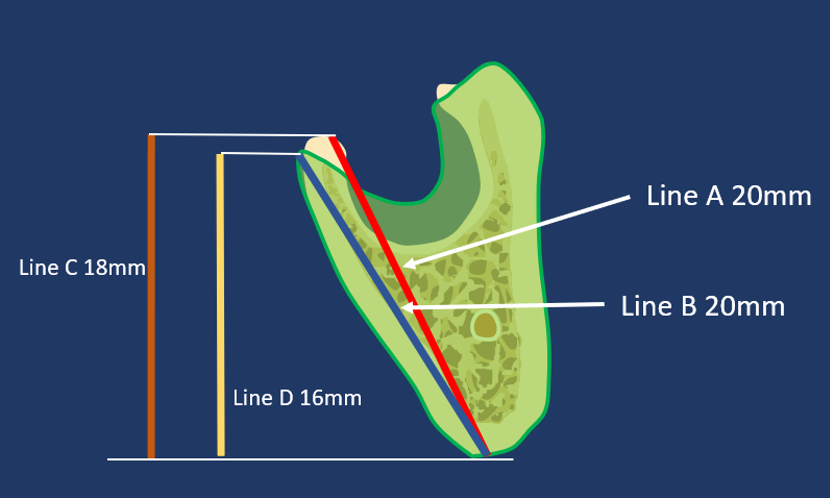

Supplement: Supplementary file 1 [file DataSheet_1.docx]
