# Supplementary material for: Alveolar ridge preservation in post-extraction sockets using concentrated growth factors: a split-mouth, randomized, controlled clinical trial
Source: Front Endocrinol (Lausanne). 2023 May 17;14:1163696. doi: 10.3389/fendo.2023.1163696 (PMC10231034; doi:10.3389/fendo.2023.1163696)
Supplement: Supplementary file 2 [file Table_1.docx]

**Table S1**; Demographic features of participants in this split-mouth study.

| Variable | Value |
| --- | --- |
| **Age,** years  Mean±SD | 25±0.5 |
| (Min-Max) | (19-35) |
| **Gender,** n (%)  Male | 30±100  16 (53.3%) |
| Female | 14 (46.7%) |
| **Impaction type,** n (%) |  |
| Horizontal | 20 (66.6%) |
| Vertical | 10 (33.7%) |
