# Supplementary material for: Alveolar ridge preservation in post-extraction sockets using concentrated growth factors: a split-mouth, randomized, controlled clinical trial
Source: Front Endocrinol (Lausanne). 2023 May 17;14:1163696. doi: 10.3389/fendo.2023.1163696 (PMC10231034; doi:10.3389/fendo.2023.1163696)
Supplement: Supplementary file 3 [file Table_2.docx]

**Table S2**; Intraclass correlation coefficient test (ICC) reliability of socket dimensions.

| **Socket dimensions** | **ICC** |
| --- | --- |
| **Buccal wall height**(mm)  Immediately after surgery  3^rd^ month after surgery | 0.94  0.96 |
| **Lingual wall height**(mm)  Immediately after surgery  3^rd^ month after surgery | 0.95  0.94 |
| **Alveolar bone width**(mm)  Immediately after surgery  3^rd^ month after surgery | 0.97  0.96 |
| **Bone density**  -Coronal half  Immediately after surgery  3^rd^ month after surgery | 0.93  0.92 |
| **Bone density**  -Apical half  Immediately after surgery  3^rd^ month after surgery | 0.92  0.91 |
| **Socket surface area**  Immediately after surgery  3^rd^ month after surgery | 0.96  0.95 |

Millimetre (mm)
